# SARS-CoV-2 spike protein binds to bacterial lipopolysaccharide and boosts proinflammatory activity

**DOI:** 10.1093/jmcb/mjaa067

**Published:** 2020-12-09

**Authors:** Ganna Petruk, Manoj Puthia, Jitka Petrlova, Firdaus Samsudin, Ann-Charlotte Strömdahl, Samuel Cerps, Lena Uller, Sven Kjellström, Peter J Bond, and Artur Schmidtchen

**Affiliations:** 1 Division of Dermatology and Venereology, Department of Clinical Sciences, Lund University, SE-22184 Lund, Sweden; 2 Bioinformatics Institute (BII), Agency for Science, Technology and Research (A*STAR), Singapore 138671, Singapore; 3 Unit of Respiratory Immunopharmacology, Department of Experimental Medicine, Lund University, SE-22184 Lund, Sweden; 4 Division of Mass Spectrometry, Department of Clinical Sciences, Lund University, SE-22184 Lund, Sweden; 5 Department of Biological Sciences, National University of Singapore, Singapore 117543, Singapore; 6 Copenhagen Wound Healing Center, Bispebjerg Hospital, Department of Biomedical Sciences, University of Copenhagen, DK-2400 Copenhagen, Denmark; 7 Dermatology, Skåne University Hospital, SE-22185 Lund, Sweden

**Keywords:** COVID-19, SARS-CoV-2, spike protein, lipopolysaccharide, inflammation, aggregation, metabolic syndrome

## Abstract

There is a link between high lipopolysaccharide (LPS) levels in the blood and the metabolic syndrome, and metabolic syndrome predisposes patients to severe COVID-19. Here, we define an interaction between SARS-CoV-2 spike (S) protein and LPS, leading to aggravated inflammation *in vitro* and *in vivo*. Native gel electrophoresis demonstrated that SARS-CoV-2 S protein binds to LPS. Microscale thermophoresis yielded a *K*_D_ of ∼47 nM for the interaction. Computational modeling and all-atom molecular dynamics simulations further substantiated the experimental results, identifying a main LPS-binding site in SARS-CoV-2 S protein. S protein, when combined with low levels of LPS, boosted nuclear factor-kappa B (NF-κB) activation in monocytic THP-1 cells and cytokine responses in human blood and peripheral blood mononuclear cells, respectively. The *in vitro* inflammatory response was further validated by employing NF-κB reporter mice and *in vivo* bioimaging. Dynamic light scattering, transmission electron microscopy, and LPS-FITC analyses demonstrated that S protein modulated the aggregation state of LPS, providing a molecular explanation for the observed boosting effect. Taken together, our results provide an interesting molecular link between excessive inflammation during infection with SARS-CoV-2 and comorbidities involving increased levels of bacterial endotoxins.

## Introduction

Coronaviruses are a group of enveloped positive-stranded RNA viruses that consist of four structural proteins including spike (S) glycoprotein (here denoted S protein), envelope (E) protein, membrane (M) protein, and nucleocapsid (N) protein (Felsenstein et al., 2020). S protein is the most important surface protein of coronavirus including SARS-CoV-2, which can mediate the entrance to human respiratory epithelial cells by interacting with the cell surface receptor angiotensin-converting enzyme 2 (Wan et al., 2020). COVID-19 disease is associated with a major inflammatory component. Increased cytokine and chemokine production in response to virus infection has been the focus of several recent investigations, and patient morbidity and mortality are mainly caused by the severe systemic inflammation and acute respiratory distress syndrome (ARDS) affecting these patients (Hariri and Hardin, 2020; Whyte et al., 2020), although differences in ARDS disease phenotypes are noticed (Li and Ma, 2020).

ARDS is a general systemic inflammatory reaction common for many disease states, such as pneumonia, severe infection, sepsis, burns, and severe trauma. During ARDS, activation of toll-like receptors (TLR), such as TLR4 via lipopolysaccharide (LPS) stimulation, induces an initial systemic proinflammatory phase characterized by a massive release of cytokines, acute phase proteins, and reactive oxygen species (Rittirsch et al., 2008; van der Poll and Opal, 2008). Additionally, activation of proteolytic cascades, like the coagulation and complement system, takes place in combination with impaired fibrinolysis and consumption of coagulation factors and other mediators (Rittirsch et al., 2008; van der Poll and Opal, 2008). Clinical symptoms of patients with ARDS therefore in many ways correspond to the pathophysiology seen during severe COVID-19 disease. There is a well-known and established link between high LPS levels in the blood and metabolic syndrome (Awoyemi et al., 2018) as well as obesity (Vors et al., 2015). Moreover, recent evidence shows that patients with metabolic syndrome are at risk of developing severe COVID-19 disease and ARDS. However, whether LPS plays a role in the pathogenesis of COVID-19 *per se* is at present unknown.

The above clinical and pathogenetic clues prompted us to investigate possible connections between LPS and SARS-CoV-2 S protein from a structural as well as functional perspective. Using electrophoresis under native conditions and microscale thermophoresis (MST) combined with computational modeling and all-atom MD simulations, we indeed found that SARS-CoV-2 S protein binds to LPS. The protein also boosted inflammatory responses when combined with low levels of LPS in monocytic THP-1 cells, human blood, and peripheral blood mononuclear cells (PBMCs) isolated from human blood. In nuclear factor-kappa B (NF-κB) reporter mice, SARS-CoV-2 S protein significantly increased the inflammatory response in conjunction with ultra-low, threshold levels of LPS. Finally, biophysical analyses combined with electron microscopy studies showed that SARS-CoV-2 S protein affects the aggregation state of LPS.

## Results

### SARS-CoV-2 S protein sequence and endotoxin content

2019-nCoV full-length His-tagged S protein (R683A, R685A), composed of the S sequence Val 16‒Pro 1213, was produced in HEK293 cells and 1 µg was analyzed on SDS–PAGE followed by staining with Coomassie brilliant blue (Supplementary Figure S1A). The results identified a major band of ∼180‒200 kDa. Although the protein has a predicted molecular weight of 134.6 kDa, the result is compatible with the expected mass due to glycosylation. Next, the band was cut off from the gel and analyzed by liquid chromatography tandem mass spectrometry (LC–MS/MS) (Supplementary Figure S1B). A total of 110 peptides covered 56% of the SARS-CoV-2 S protein sequence, confirming identity. Using a limulus amebocyte lysate (LAL) assay, the LPS content in the recombinant S protein was determined to 30 fg/µg protein.

### Studies on the interaction between SARS-CoV-2 S protein and LPS

**Figure 1:**
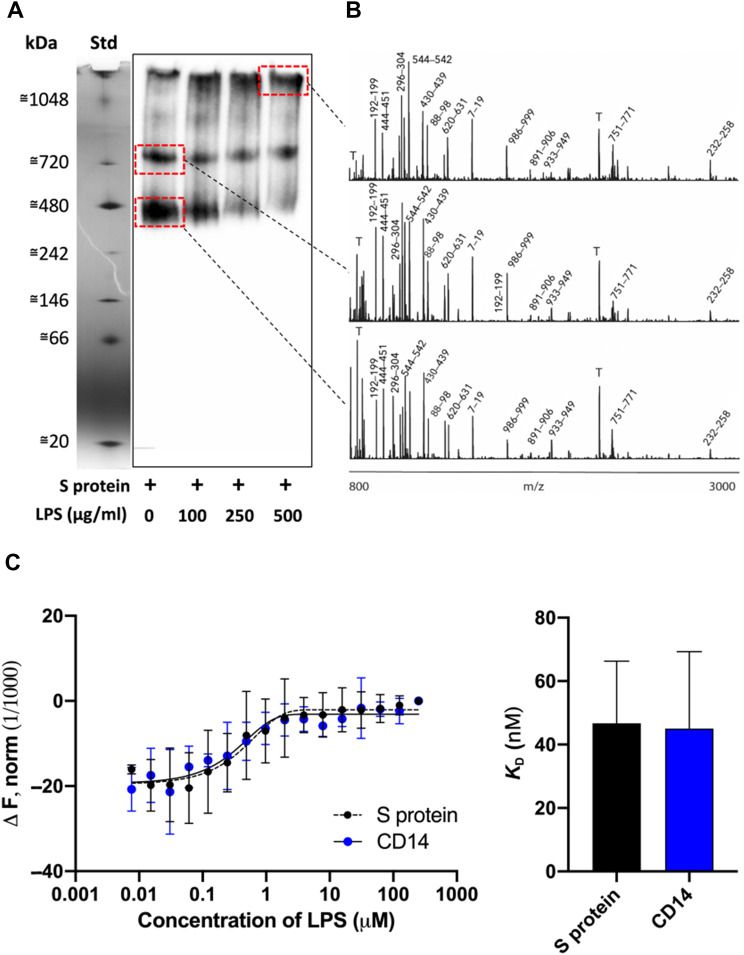
Analysis of the interaction between SARS-CoV-2 S protein and LPS *in vitro*. (**A**) SARS-CoV-2 S protein was incubated with LPS (0‒500 µg/ml), separated using BN-PAGE and detected by western blotting. One representative image of three independent experiments is shown (*n *=* *3). The marker lane is from the same gel but not transferred to the membrane. It is aligned and included for clarity. (**B**) Gel pieces corresponding to the area denoted by the dotted red squares on the western blot were cut out, in-gel digestion was performed, and the material was subjected to MALDI MS analysis. Representative high resolution MALDI mass spectra are presented. The most intense tryptic fragments obtained from S protein are denoted with the sequence numbers, and tryptic peptides from the autodigestion of trypsin are denoted with T. (**C**) MST assay quantifying SARS-CoV-2 S protein interaction with LPS. CD14 was used as positive control. *K*_D_ constant for S protein = 46.7 ± 19.7 nM vs. CD14 = 45 ± 24.3 nM was determined from MST analysis. Mean ± SD values of six measurements are shown (*n* = 6).

Native gel electrophoresis is used as a tool to assess structural differences in proteins, but alterations were also induced by binding to external ligands. We therefore decided to study the migration of S protein alone or in presence of increasing doses of *Escherichia coli* LPS (Figure 1A). Under the conditions used, S protein migrated at the molecular mass range of 400‒500 kDa. A second higher molecular 700‒800 kDa band of less intensity was, however, observed. Addition of increasing doses of LPS indeed yielded a shift in the migration of S protein, with a reduction of particularly the 400‒500 kDa band and an increase of high-molecular weight material not entering the gel. MS of the excised protein bands was then performed. The results verified that the bands of 400‒500 and 700‒800 kDa were composed of S protein. S protein was also identified in the high-molecular weight fraction found in the samples incubated with LPS (Figure 1B). Analogously, MST, a highly sensitive technique probing interactions between components in solution, demonstrated interactions of fluorescence-labelled S protein with *E. coli* LPS, with a *K*_D_ of 46.7 ± 19.7 nM (Figure 1C). For control in these experiments, we used the well-known human LPS receptor CD14, which exhibited a *K*_D_ of 45.0 ± 24.3 nM to LPS. In order to gain more information on the interaction specificity, we evaluated binding of S protein to the lipid part of LPS, Lipid A (Supplementary Figure S2A), as well as other microbial agonists (Supplementary Figure S2B). S protein was found to interact with Lipid A (Supplementary Figure S2A) and also LPS from *Pseudomonas aeruginosa*, whereas no shift in the migration was observed after addition of lipoteichoic acid (LTA), peptidoglycan (PGN), or zymosan (Supplementary Figure S2B). Having shown a ligand specificity for SARS-CoV-2 S protein, we next explored whether LPS could bind to the related SARS-CoV S protein. The results indeed showed that addition of LPS yielded an apparent migration shift also for this protein (*P* < 0.05). For control, we used the human protein prothrombin, which showed no shift in migration (*P* = 0.98). Taken together, using two independent methods probing molecular interactions, a specific binding of LPS to SARS-CoV-2 S protein was identified. Notably, the affinity of LPS to SARS-CoV-2 S protein was in the range of the one observed for LPS binding to the human receptor CD14. Moreover, the related SARS-CoV S protein also bound to LPS.

### Computational modeling and simulations of SARS-CoV-2 S protein and LPS

**Figure 2:**
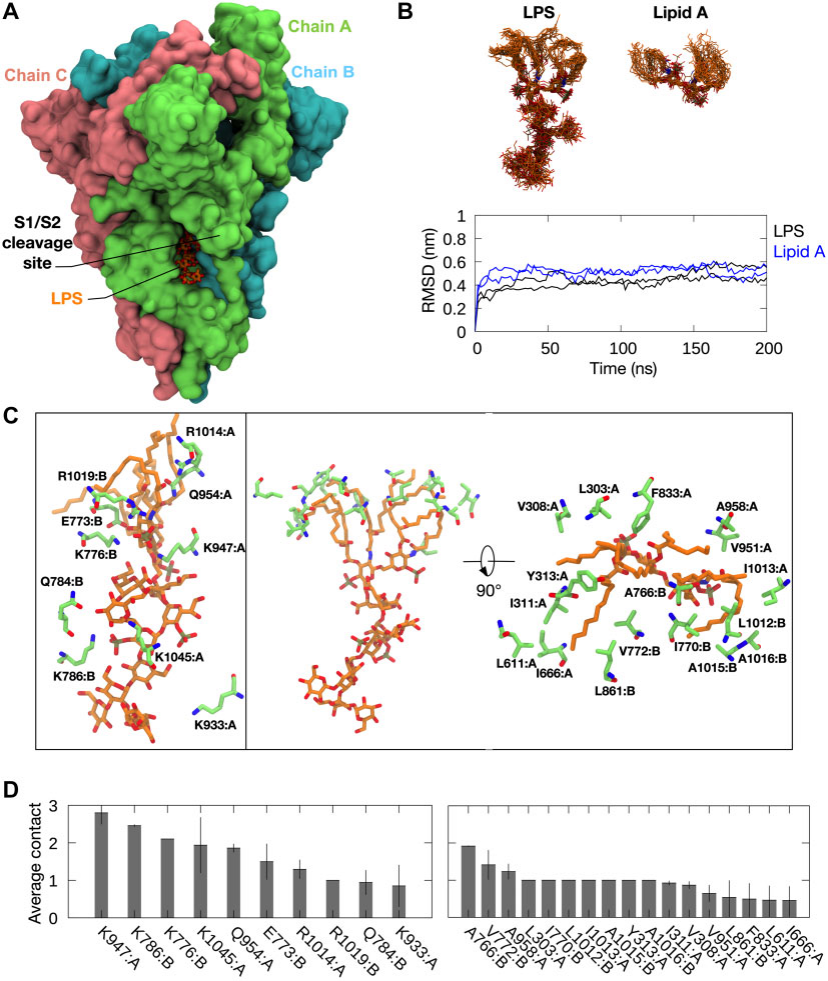
Predicted LPS-binding site on SARS-CoV-2 S protein. (**A**) The proposed binding site of LPS (orange; stick representation) on S ECD (green, pink, cyan; surface representation). The S1/S2 furin cleavage site is labelled. (**B**) Top: snapshots of LPS and Lipid A overlaid on to one another, each taken every 10 ns from two independent 200 ns simulations of S ECD with LPS and Lipid A bound to the predicted binding site. Bottom: RMSD of LPS and Lipid A throughout the simulations. (**C**) S protein residues interacting with the LPS headgroup (left) and lipid tails (right) during these simulations. (**D**) The average number of contacts made by these residues with LPS headgroup (left) and lipid tails (right). Error bars show standard deviations between two repeat simulations.

To predict the binding site of LPS on the SARS-CoV-2 S protein, we performed flexible docking using *E. coli* rough LPS and Lipid A. These two ligands were docked onto the structures of S ectodomain (ECD) in the open and close conformational states (Cai et al., 2020; Wrapp et al., 2020) using the Vina-Carb docking program, a variation of AutoDock Vina that incorporates the flexibility of glycosidic linkages in oligosaccharide into its scoring function (Nivedha et al., 2016). A total of 40 docking poses were generated (Supplementary Figure S3) and each pose was assessed by the presence of basic residues stabilizing the phosphate groups and hydrophobic residues around the lipid tails of LPS and Lipid A. Interestingly, the most frequently sampled binding site (21 out of 40 poses) was found within the proximity of the S1/S2 furin cleavage site (Hoffmann et al., 2020). The binding of LPS and Lipid A was predicted at a groove between two protomers (Figure 2A), whereby a ladder of basic residues surrounded the phosphate and sugar moieties, while a hydrophobic pocket at the top of the grove accommodated the lipid tails. To determine the binding stability of LPS and Lipid A at this site, we performed all-atom MD simulations of S ECD with bound LPS and Lipid A. In all simulations, both LPS and Lipid A bound stably to the proposed binding site, with associated lipid root mean square deviation (RMSD) values reaching a plateau at ∼0.4 nm after 50 ns or less (Figure 2B). In simulations with LPS, the Lipid A residue and adjacent core sugars displayed little movement, while the terminal core sugars showed some degree of flexibility as they were less buried within the binding site. Contact analysis indicated that the interactions stabilizing LPS and Lipid A binding were contributed from two chains of the S ECD trimer, with most of the residues located in the S2 subunit of the protein. The phospho-GlcNac moieties of the Lipid A residue formed ionic interactions with K776, K947, and R1019, while the phosphorylated core sugars were stabilized by salt bridge interactions with K786 and K1045 (Figure 2C and D). Sodium ions made intermittent contacts with some of the phosphate groups, suggesting a potential role of cations in LPS binding. All of the six lipid tails occupied a pocket made up of hydrophobic residues including V772, I666, L611, A647, and Y313. Overall, the coordination of LPS with S protein at this proposed binding site is similar to that with LPS receptors or coreceptors including CD14 and MD-2, whereby the lipid tails are buried deep within a hydrophobic pocket, while the phosphorylated sugar moieties are more exposed to the solvent area and form hydrophilic interactions with basic and polar residues located at the opening area (Park et al., 2009; Huber et al., 2018; Saravanan et al., 2018). The flexible loop housing the S1/S2 furin cleavage site made intermittent interactions with LPS, specifically with the terminal core sugars. Structural alignment to the S protein from SARS-CoV (Yuan et al., 2017) revealed that, indeed, most of the residues forming the proposed binding site are conserved (Supplementary Figure S4), in agreement with the interaction observed between SARS-CoV S protein and LPS during native gel electrophoresis (Supplementary Figure S2C) and further validating this location as the likely site of a high-affinity LPS interaction.

### Effects of SARS-CoV-2 S protein on LPS-induced responses in vitro

**Figure 3:**
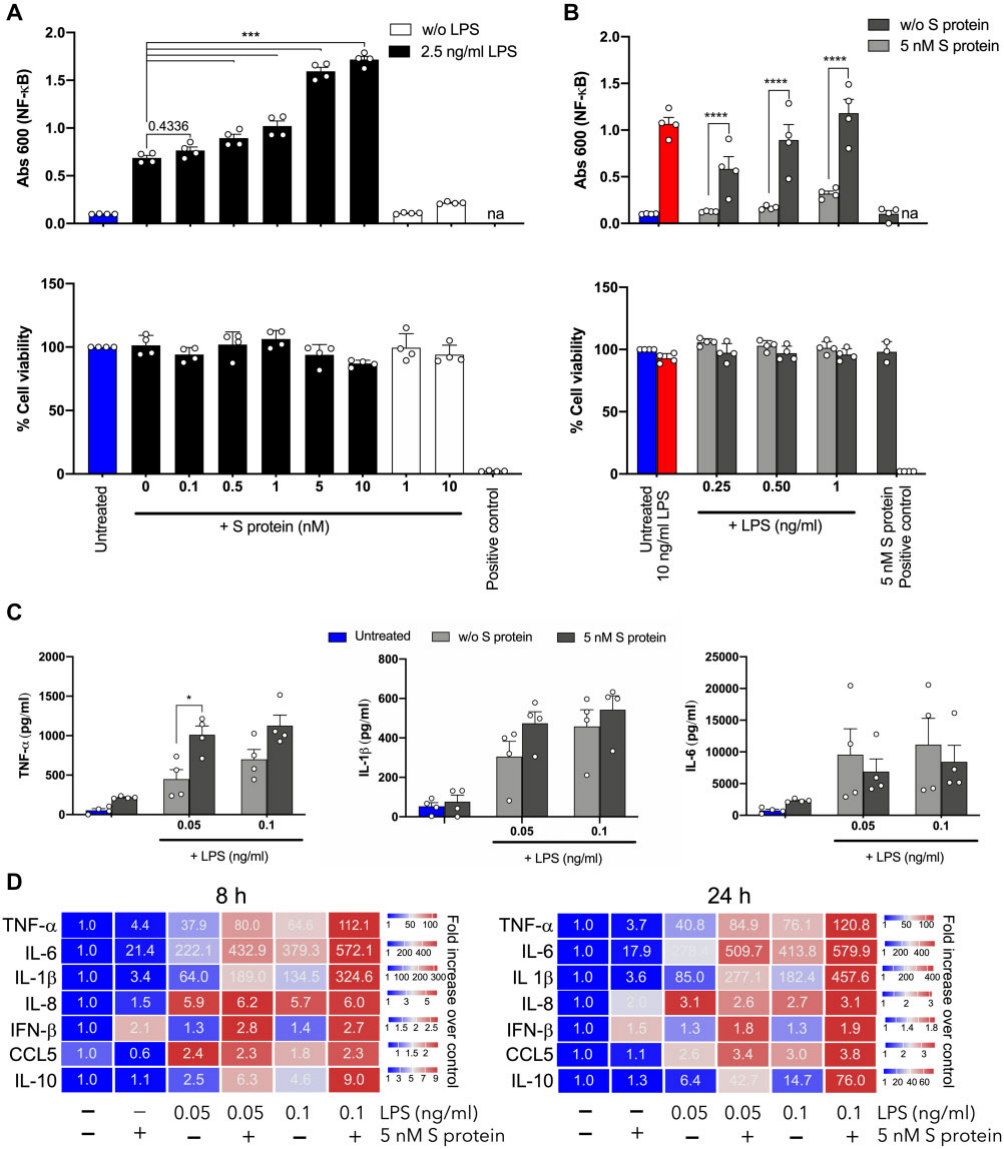
Effects of SARS-CoV-2 S protein on LPS-induced responses in THP-1 cells. (**A** and **B**) THP-1-XBlue-CD14 cells were treated with increasing concentrations of SARS-CoV-2 S protein (0‒10 nM) and a constant dose of LPS (2.5 ng/ml) (**A**) or with increasing doses of LPS (0.25‒1 ng/ml) and constant amount of S protein (5 nM) (**B**). MTT viability assay for analysis of toxic effects of S protein and LPS on THP-1 cells is shown in lower panels. (**C**) Cytokine analysis of the blood collected from healthy donors at 24 h after treatment with S protein with or without 0.05 and 0.1 ng/ml LPS. Untreated blood was used as a control. The mean ± SEM (NF-κB and blood assays) or mean ± SD (MTT assay) values of four independent experiments performed in duplicate are shown (*n *= 4). *P ≤ 0.05, ***P ≤ 0.001, ****P ≤ 0.0001, determined using two-way ANOVA with Sidak’s multiple comparisons test (NF-κB and blood assays) or one-way ANOVA with Dunnett’s multiple comparison test (MTT assay). na, not analyzed; w/o, without. (**D**) Heatmap showing cytokines released from PBMCs, isolated from three different donors and treated with SARS-CoV-2 S protein (5 nM) and increasing doses of LPS (0.05‒0.1 ng/ml) for 8 and 24 h. The cytokines were detected by Luminex multiplex bead assay. Color and values in each box represent mean values of fold increase over untreated cells (*n* = 3).

LPS effects depend on specific interactions with components of innate immunity such as LPS-binding protein (LBP), culminating in transfer of LPS from CD14 to toll-like receptor 4 (TLR4) and its coreceptor MD-2 on the cell surface, leading to activation of downstream inflammatory responses (Huber et al., 2018). In order to probe whether the presentation and hence activity of LPS were altered by the interaction with SARS-CoV-2 S protein, we decided to study the proinflammatory effects of S protein with or without LPS using THP1-XBlue-CD14 cells. After 18–24 h of incubation, NF-κB/AP-1 activation and cell metabolic activity was determined. In order to assess potential changes in the LPS response, we used a low dose of LPS of 2.5 ng/ml, which is a dose that regularly yields about 20%‒40% of the maximal response elicited by 100 ng/ml LPS. Addition of S protein at increasing concentrations resulted in a gradual and significant increase in NF-κB/AP-1 activation (Figure 3A). It was also observed that SARS-CoV-2 S protein alone did not induce any significant increase in NF-κB/AP-1 activation at the concentrations used. Of relevance for the above is that the endogenous levels of LPS in the S protein preparation were negligible, as they were in the order of 100–1000 lower than the threshold level required for NF-κB activation. In general, patients with a systemic inflammatory response such as seen in sepsis show increased levels of LPS in plasma, with levels ranging from 0.1 to 1 ng/ml (Opal et al., 1999). In order to mimic those LPS levels, we therefore determined the response of the THP-1 cells to doses ranging from 0.25 to 1 ng/ml LPS, with or without the presence of 5 nM S protein. It was observed that NF-κB activation was significantly boosted even at those low doses of LPS. Notably, LPS at 0.25 ng/ml, which alone did not induce a significant increase of NF-κB activation, yielded a significant response together with SARS-CoV-2 S protein. It was also observed that LPS at doses of 0.5–1 ng/ml, combined with SARS-CoV-2 S protein, yielded response levels produced by 10 ng/ml LPS (Figure 3B). In line with the LPS-binding results (Supplementary Figure S2C), a similar boosting effect on the LPS response was also observed for SARS-CoV S protein (Supplementary Figure S5). In these studies, cell viability was regularly measured by MTT assay, and no significant toxic effects were detected (Figure 3A and B; Supplementary Figure S5). Using human blood, we observed a similar increase of the LPS response. Again, particularly ultra-low levels of LPS, 50 pg/ml, showed boosted tumor necrosis factor alpha (TNF-α) levels together with S protein (Figure 3C). These results translated the boosting by SARS-CoV-2 S protein to a physiologically relevant blood milieu, mimicking the raised LPS levels found in conditions characterized by endotoxinemia (Table 1). Given the complex blood environment and the fact that individual cytokines show a temporal variation as well as interdependence (DeForge and Remick, 1991; Agarwal et al., 1995; Debey et al., 2004; Liu et al., 2018), we next decided to use human PBMCs for the subsequent detailed analyses of inflammatory responses at an early or late time point after stimulation. The results showed that the combination of ultra-low levels of LPS and SARS-CoV-2 S protein yielded significant boosting of TNF-α and interleukin-6 (IL-6) at both time points analyzed (8 and 24 h), whereas the observed increases of IL-1β and IL-10 did not reach statistical significance (Figure 3D; Supplementary Table S1). IL-8 was increased relative to controls, yielding similar levels at both time points, after stimulation with 50 or 100 pg LPS alone. SARS-CoV-2 S protein at 5 nM did neither induce IL-8 alone nor boost the LPS response. Notable was that IFN-β was significantly increased by SARS-CoV-2 S protein alone at both time points, irrespective of the addition of LPS (Figure 3D; Supplementary Table S1). Taken together, these results demonstrate that SARS-CoV-2 S protein increases LPS responses *in vitro* in monocytic cells, human blood, and PBMCs and, in particular, the activation by low/threshold levels of LPS is boosted several-fold by the addition of SARS-CoV-2 S protein. Moreover, the boosting was particularly observed for cytokines directly dependent on NF-κB activation, thus representing the proximal LPS-dependent cytokine response (DeForge and Remick, 1991; Liu et al., 2018).

**Table 1:** Diseases involving endotoxins and links to COVID-19.

Disease/indication	LPS levels in plasma or serum	References	Bacterial influence	References	COVID-19 and risk for severe disease	References
Metabolic syndrome	47‒96 EU/ml	Cani et al. (2007); Lassenius et al. (2011); Awoyemi et al. (2018)	Dysbiosis	Dabke et al. (2019)	12%	Costa et al. (2020); Marhl et al. (2020)
COPD	Unknown	de Oliveira et al. (2019)	Microbial colonization	Nys et al. (2000)	1%‒3%	Alqahtani et al. (2020)
Inflammatory bowel syndrome (IBD)	44.41 ± 89.44 pg/ml	Pastor Rojo et al. (2007); Guo et al. (2015)	Dysbiosis	Tamboli et al. (2004); Zuo and Ng (2018)	Unknown	Bezzio et al. (2020)
Kawasaki disease	Unknown	Tsujimoto et al. (2001)	Unknown	—	Unknown	Alizargar (2020)
Periodontitis	0.89 ± 2.90 ng/ml	Paju et al. (2006)	Microbial colonization	Bui et al. (2019)	Unknown	Sahni and Gupta (2020)

### Effects of SARS-CoV-2 S protein on endotoxin responses in an experimental mouse model

**Figure 4:**
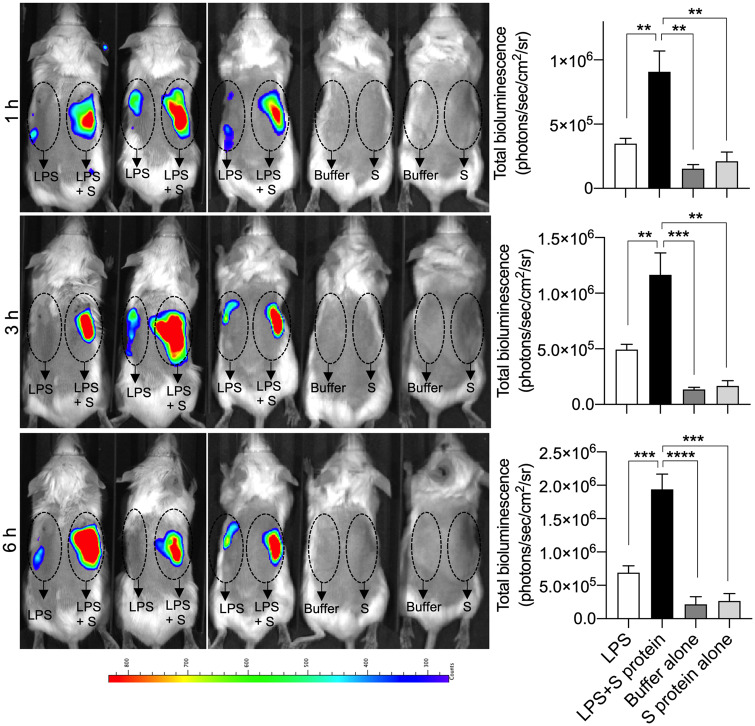
SARS-CoV-2 S protein combined with LPS boosts inflammation in NF-κB reporter mice. For *in vivo* inflammation imaging in NF-κB reporter mice, LPS alone or in combination with SARS-CoV-2 S protein (S) was subcutaneously deposited on the left or right side, respectively, on the back of transgenic BALB/c Tg(NF-κB-RE-luc)-Xen reporter mice. Noninvasive *in vivo* bioimaging of NF-κB reporter gene expression was performed using the IVIS Spectrum system. Representative images show bioluminescence at 1, 3, and 6 h after subcutaneous deposition. A bar chart shows measured bioluminescence intensity emitted from these reporter mice. Dotted circles represent area of subcutaneous deposition and region of interest for data analysis. Data are presented as mean ± SEM (*n *=* *5 mice for LPS group, 5 mice for LPS and S protein group, 3 mice for buffer control, and 3 mice for S protein control). *P*-values were determined using a one-way ANOVA with Holm-Sidak posttest. **P ≤ 0.01, ***P ≤ 0.001, ****P ≤ 0.0001.

In an experimental animal model, we wanted to simulate a situation of localized endotoxin-induced inflammation. In previous models, we utilized 25 µg LPS injected subcutaneously, a dose level that yielded a robust and significant LPS response (Puthia et al., 2020). In this modified model, similar to the strategy described above on the THP-1 cells, we employed a low threshold level comprising 2 µg LPS, which was injected subcutaneously with or without 5 µg S protein. Using mice reporting NF-κB activation, we indeed found that the addition of S protein significantly increased the inflammatory response (Figure 4). SARS-CoV-2 S protein alone at the dose of 5 µg did not yield any significant inflammatory response. Apart from a strongly increased response by the LPS and SARS-CoV-2 S protein combination, we also observed that the LPS‒S protein mix resulted in a prolonged NF-κB response during the time period studied. Taken together, the results demonstrated that SARS-CoV-2 S protein also retains its boosting effect in conjunction with LPS in a subcutaneous model of endotoxin-driven inflammation.

### Effects of SARS-CoV-2 S protein on LPS aggregation

**Figure 5:**
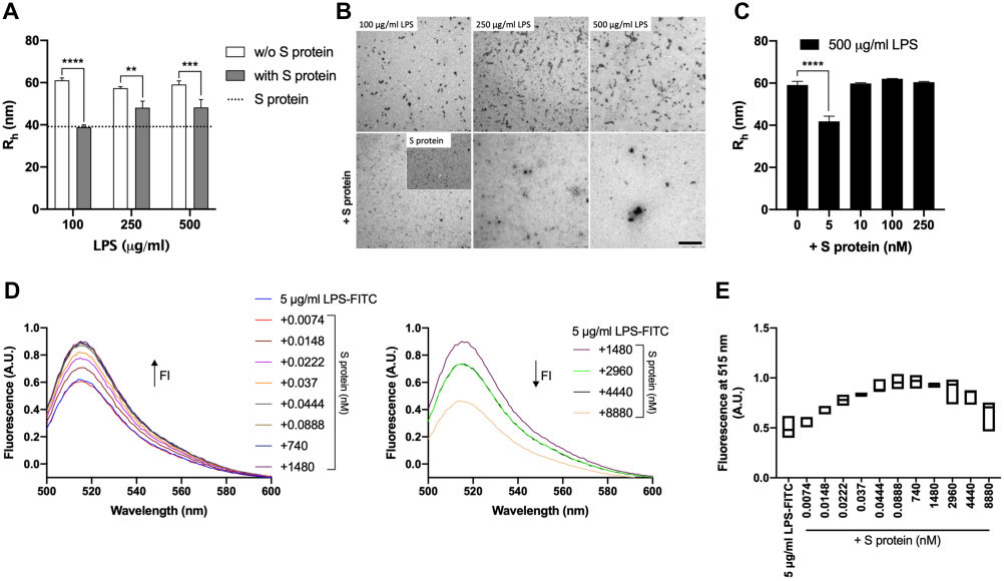
Effects of different doses of SARS-CoV-2 S protein on biophysical properties of LPS. (**A** and **B**) Increasing concentrations of LPS (100‒500 µg/ml) alone or with S protein (1.48 µM) were incubated for 30 min at 37°C and then analyzed by DLS (**A**) and TEM (**B**). (**C**) LPS (500 µg/ml) alone or with S protein (5‒250 nM) was incubated for 30 min at 37°C and hydrodynamic radii of the particles in solution were measured by DLS. For DLS, the data are presented as mean ± SEM (*n* = 3). *P*-values were determined using a one-way ANOVA with Sidak’s multiple comparisons test. **P ≤ 0.01, ***P ≤ 0.001, ****P ≤ 0.0001. For TEM, one representative picture per each condition is shown (*n* = 3). (**D** and **E**) The fluorescence intensity of LPS-FITC (5 µg/ml) alone or with different concentrations of S protein (0.0074‒8880 nM) was measured by recording the emission fluorescence spectra between 500 and 600 nm, following excitation at 488 nm. Graphs with spectra are a representative result of three independent experiments (*n* = 3). The change in fluorescence is indicated by an arrow. (**E**) The fluorescence at 515 nm of FITC-LPS plotted with respect to the concentrations of the protein is presented as floating bars (min to max) with line at median (*n* = 3). A.U., arbitrary units.

The finding that SARS-CoV-2 S protein both binds to and boosts LPS responses prompted us to further investigate the interaction and its consequences on the organization of LPS micelles. Increasing doses of LPS alone or with a constant amount of S protein were incubated and the hydrodynamic radii of the particles in the solution were measured by dynamic light scattering (DLS). The size of LPS particles was found to be ∼60 nm, in agreement with previous data (Petrlova et al., 2017), and moreover, they were not affected by the concentration of LPS in the dose range studied (Figure 5A). Incubation of 100 µg/ml of LPS with SARS-CoV-2 S protein, yielded a significant reduction of the hydrodynamic radii of the particles in solution. Notably, the aggregate size was similar to the one observed in the sample with SARS-CoV-2 S protein alone, suggesting a complete dispersion of LPS aggregates by S protein. A less pronounced, albeit significant, disaggregation was observed when the LPS concentration was increased to 250 and 500 µg/ml, respectively (Figure 5A). Next, transmission electron microscopy (TEM) was employed in order to further characterize the LPS micelles. Corresponding to the DLS data, a marked disaggregating effect on the LPS micelles was detected using 100 µg/ml LPS (Figure 5B). In the samples with 250 and 500 µg/ml LPS, the appearance of larger aggregates was noted, suggestive of the LPS–SARS-CoV-2 S protein complexes identified by blue native (BN)-PAGE (Figure 1A). In order to further study the dose-dependence of the disaggregation and aggregation processes, we incubated 500 µg/ml LPS with increasing doses of S protein (5‒250 nM) and analyzed the resulting particles hydrodynamic radii by DLS (Figure 5C). The results showed that 5 nM of SARS-CoV-2 S protein disaggregated LPS, whereas addition of S protein at higher levels induced aggregation. The data obtained with DLS was further confirmed by studying complexes of fluorescein-labelled LPS (LPS-FITC) in the presence of increasing concentrations of SARS-CoV-2 S protein (Figure 5D and E). A gradual increase in fluorescence was observed by adding subnanomolar amounts of S protein, indicating a reduction in fluorescein self-quenching due to S protein-induced disaggregation of LPS (Figure 5D, left panel). With increasing S protein concentrations, the fluorescence level was increased up to a maximum level, indicating a complete dispersion of LPS aggregates. Using higher levels of S protein, however, a decrease in fluorescence intensity of LPS-FITC was noticed, indicating subsequent aggregation (Figure 5D, right panel). Plotting the fluorescence intensity at 515 nm as function of different concentrations of S protein demonstrated the dose-dependence of the disaggregation and aggregation processes (Figure 5E). In summary, these data show the dynamic and dose-dependent interactions within SARS-CoV-2 S protein–LPS complexes. Notably, SARS-CoV-2 S protein induced a marked disaggregation of LPS at subnanomolar to nanomolar levels.

## Discussion

Here, we demonstrate a previously unknown interaction between SARS-CoV-2 S protein and LPS, leading to a boosting of proinflammatory actions *in vitro* as well as *in vivo*. These results on the synergism between LPS and S protein have clinical and therapeutic importance, as this could give new insights in the comorbidities that may increase the risk for severe COVID-19 disease and ARDS, its pathogenetic steps, as well as provide new therapeutic targets.

**Figure 6:**
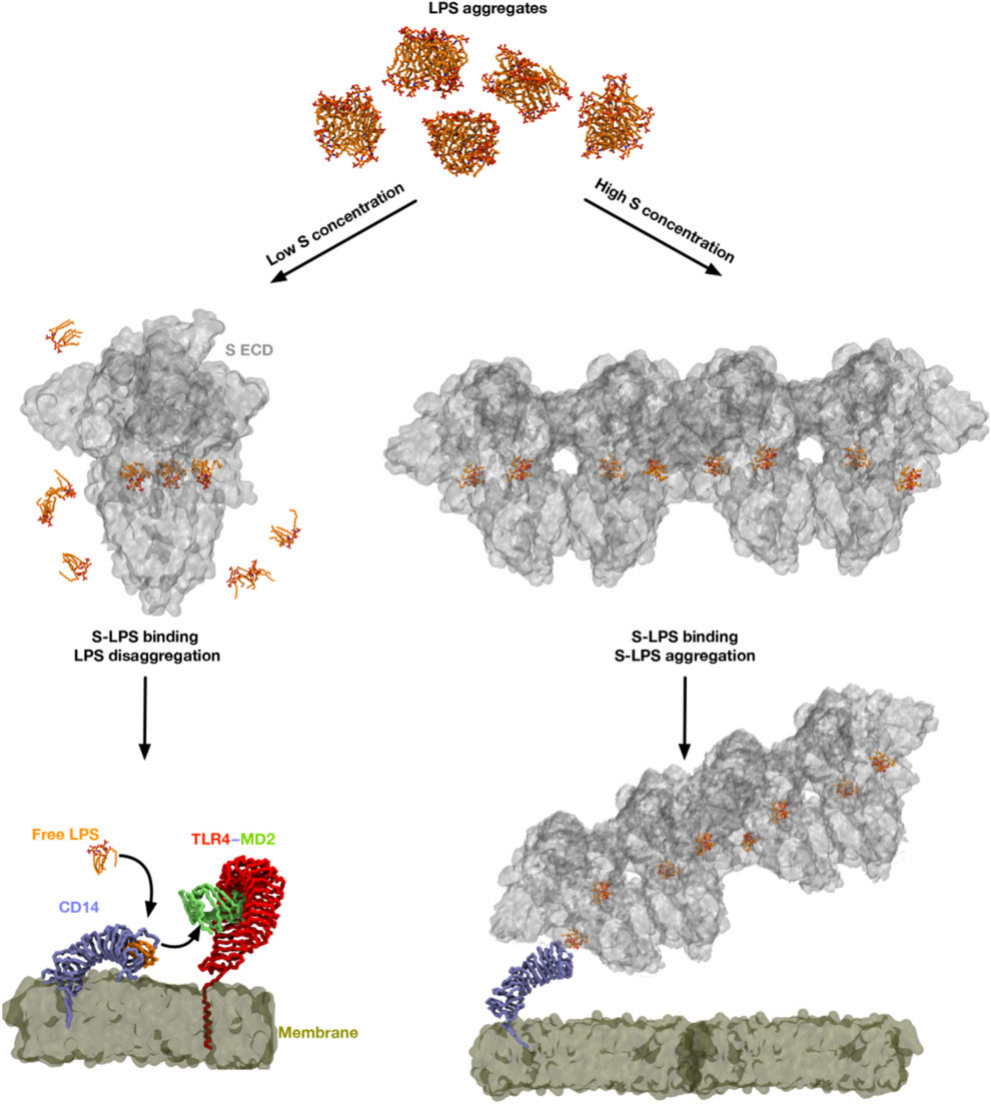
Proposed proinflammatory mechanism of SARS-CoV-2 S protein. In the absence of S protein, LPS micelles are present. At low S protein concentrations, some LPS molecules bind to S protein resulting in disaggregation of the LPS aggregates. Free LPS molecules are then able to bind to the LPS receptor, CD14, before being transferred to the TLR4/MD2 complex, which activates downstream signaling. At high S protein concentrations, most LPS molecules are bound to the S protein, and the S protein‒LPS complex then forms large aggregates [modelled based on S protein dimer of trimers (Bangaru et al., 2020)], which may promote inflammatory responses.

The molecular mechanism underlying the observed boosting of inflammation by SARS-CoV-2 S protein was shown to be dependent on specific and distinct interactions between S protein and LPS, leading to changes in the biophysical state of LPS. Thus, MST analysis measuring the interaction between S protein and LPS yielded a *K*_D_ in the nM range, which indicates a high-affinity binding in the same range as observed for the interaction between LPS and CD14 (Figure 1C) and, interestingly, also LBP (Ryu et al., 2017). Moreover, electrophoresis under native conditions confirmed the SARS-CoV-2 S protein‒LPS binding. It is of note that the binding was also observed for Lipid A, the core of the LPS molecule, whereas the TLR agonists LTA, PGN, and zymosan did not interact with SARS-CoV-2 S protein. As Lipid A is a common structure in all endotoxin-producing Gram-negative bacteria, the results therefore imply that the findings with *E. coli* can be generalized to endotoxins from other Gram-negative bacteria. In support of this assumption was the finding that LPS from the pathogen *P. aeruginosa* also bound to SARS-CoV-2 S protein (Supplementary Figure S2B). Computational modeling and simulations of SARS-CoV-2 S protein and LPS and Lipid A elegantly substantiated the experimental results, and the interaction site for LPS and Lipid A was predicted at a groove between two protomers (Figure 2A). Importantly, all-atom MD simulations of the extracellular domain of SARS-CoV-2 S protein with bound LPS and Lipid A demonstrated that both LPS and Lipid A bound stably to the proposed binding site, well in agreement with the observed high-affinity binding to LPS demonstrated by MST. Interestingly, the coordination of LPS at this binding site is similar to that demonstrated for other LPS receptors including CD14 and MD-2 (Park et al., 2009; Saravanan et al., 2018). Also notable was that this groove is conserved in the related SARS-CoV S protein, which also was found to bind to LPS. Complex and sequential interactions between LPS and LBP aid in LPS transfer to CD14 and subsequent downstream NF-κB activation (Ryu et al., 2017; Huber et al., 2018), and disaggregation of LPS by LPB as well as other proteins has been reported to boost LPS proinflammatory effects in various settings (Tobias et al., 1997; Kitchens and Thompson, 2005; Bodet et al., 2007; Komatsu et al., 2016). These molecular and functional links to other LPS-binding proteins and their effects on the aggregation state of LPS prompted a series of investigations involving DLS, TEM, and LPS-FITC analyses. Together, these experiments conclusively demonstrated that SARS-CoV-2 S protein modulates the aggregation state of LPS, analogously to the effects reported for other LPS-binding proteins such as LBP (Kitchens and Thompson, 2005; Ryu et al., 2017). Further, previous reports indicate that LPS and protein aggregates can be proinflammatory (Mueller et al., 2004; Dalgediene et al., 2018). Moreover, LBP can form aggregates with LPS, particularly at a high LPS/LBP ratio (Kitchens and Thompson, 2005), providing additional links to the present findings with SARS-CoV-2 S protein. Additionally, recent cryo-EM structures of SARS-CoV-2 S protein from an advanced vaccine candidate show that S protein can form higher order oligomers, such as dimer of trimers and trimer of trimers (Bangaru et al., 2020), further supporting the observations on the formation of large S‒LPS aggregates in this study. Figure 6 summarizes the experimental and *in silico* data and depicts the proposed mode of action for the SARS-CoV-2 S protein-mediated LPS boosting effect.

As aforementioned, the observed clinically relevant link between high LPS levels in the blood and the metabolic syndrome (Awoyemi et al., 2018) and the fact that metabolic syndrome is a risk factor for developing severe COVID-19 originally prompted this study (Felsenstein et al., 2020). However, as summarized in Table 1, the clinical implications may be broader and go beyond metabolic syndrome. Notably, in patients with chronic obstructive pulmonary disease (COPD), COVID-19 infection is associated with substantial severity and mortality rates (Alqahtani et al., 2020). Therefore, a causative link between LPS derived from bacterial colonization and infection of the lungs in COPD patients and COVID-19 severity could also be proposed here. Related to this is the finding that there is a correlation between LPS levels and bacterial loads during pneumonia (Nys et al., 2000). Moreover, compared to former and never smokers with COPD, current smokers are at greater risk of severe COVID-19 complications and higher mortality rate (Alqahtani et al., 2020), and intriguingly, bacterial LPS is an active component of cigarette smoke (Hasday et al., 1999). Increased endotoxin levels are also observed in patients with inflammatory bowel disease (IBD) (Pastor Rojo et al., 2007). The observations that all these comorbidities are risk factors for severe COVID-19 lend further support for a pathogenetic link to SARS-CoV-2 infection and endotoxinemia, Moreover, intriguingly, Kawasaki disease in children, which has been reported in young COVID-19 patients (Toubiana et al., 2020) as well as in patients with SARS HCoV-NH (Esper et al., 2005), has been linked to LPS as a trigger (Tsujimoto et al., 2001). Other possible comorbidities that should be considered include periodontitis, where LPS from *Porphyromonas gingivalis* and other bacteria can reach the systemic circulation (Bui et al., 2019). Indeed, a recent hypothesis on this matter has been raised (Sahni and Gupta, 2020). All these observations on links between LPS levels and several diseases and conditions (Table 1), along with the risk for developing severe COVID-19 (Felsenstein et al., 2020), imply that measurement of endotoxin levels in COVID-19 patients could have significant diagnostic implications and be of relevance for patient management and treatment decisions. Clearly, clinical prospective studies are mandated in order to assess whether the findings from the present study can be translated to the clinical setting.

Although here disclosed binding of LPS to SARS-CoV-2 S protein is novel, the interaction between S proteins and endotoxins is not necessarily new to nature. Indeed, interactions between viruses and bacteria for the induction of severe respiratory disease have been described since the early thirties (Shope, 1931). Obviously, multiple complex and diverse inflammatory mechanisms may underlie this general finding. However, it is worth noting that recent observations from porcine animal models indeed demonstrated that infection with porcine respiratory coronavirus, a highly prevalent virus in swine populations significantly sensitizes the lungs to LPS (Van Gucht et al., 2006). Notably, the effects of separate virus or LPS inoculation were subclinical and failed to induce sustained cytokine levels, whereas the combination of the two agents significantly triggered severe respiratory disease and enhanced particularly TNF-α levels (Van Reeth et al., 2000), which are indeed relevant in the light of the present results showing boosted TNF-α levels in human blood and PBMCs with the combination of LPS and SARS-CoV-2 S protein. In agreement with this, it is also worth noting that the disease denoted ‘Shipping fever’, which affects cattle particularly in relation to stress and transports, can be triggered by a combination of bovine respiratory coronavirus (BCoV) and inhaled LPS (Saif, 2010).

In conclusion, we report a previously undisclosed interaction between the SARS-CoV-2 S protein and LPS and its link to induction of NF-κB and cytokine responses in monocytes, PBMCs, and human blood, as well as increased NF-κB responses in experimental animal models. The interaction between S protein and LPS therefore provides a new therapeutic target enabling development of drugs that can ameliorate the hyperinflammation seen during COVID-19 infection.

## Materials and methods

### Proteins

SARS-CoV-2 and SARS-CoV S proteins were produced by ACROBiosystems. The sequence of SARS-CoV-2 S protein contains amino acids Val 16‒Pro 1213 [Accession # QHD43416.1 (R683A, R685A)], whereas the sequence of SARS-CoV S protein contains amino acids Ser 14‒Pro 1195 [Accession # AAP13567.1 (R667A)]. Briefly, both full-length S proteins with His-Tag (SPN-C52H4) were expressed in human 293 cells (HEK293) and purified. The proteins were lyophilized from a 0.22-μm-filtered solution in 50 mM Tris, 150 mM NaCl, pH 7.5. Lyophilized products were reconstituted in endotoxin-free water, aliquoted, and stored at −80°C according to the manufacturer’s protocol. The purity was >85% and >95% for SARS-CoV-2 S protein and SARS-CoV S protein, respectively. Human His-Tag-CD14 (hCD14-his) was produced recombinantly in insect cells by using the Baculovirus Expression Vector System (BEVS). Since this construct is secreted, medium was centrifuged in a JLA8-1000 rotor at 8000 *g*, 20 min, 4°C, and then the supernatant was filtered with a PES 0.45 μm filter top (0.45 μm pore size). Subsequently, hCD14-his was purified on a 5-ml HisTrap Excel column (GE Healthcare) by employing ÄKTA Pure system (GE Healthcare). Eluted fractions were analyzed by precast SDS–PAGE gel (Bio-Rad) stained with BioSafe Coomassie (Bio-Rad) or subjected to western blotting. Peak fractions containing the protein of interest were pooled and digested with tobacco etch virus (TEV) protease to remove the His-Tag. After TEV digestion, the protein solution was run a second time on the HisTrap column. Fractions containing the protein were collected, pooled, and purified further on a HiLoad 26/60 Superdex 75 pg gel filtration column. At the end of purification, the purity of hCD14 was estimated to >90%. The protein was aliquoted and stored at −80°C before use. Human prothrombin was obtained from Innovative Research.

### LAL assay

The content of endotoxin in 1 µg purified SARS-CoV-2 S protein was analyzed using a commercially available Pierce™ Chromogenic Endotoxin Quant Kit (Thermo-Fisher) according to the manufacturer’s protocol with small modifications. In particular, the standard curve was done with LPS from *E. coli* (Sigma) in the range of 0.01‒10 pg/ml. All samples were prepared in endotoxin-free tubes kept in a thermoblock set to 37°C. At the end of the incubation, 150 µl of each sample was transferred to 96-well plates and analyzed for absorbance at 405 nm using a spectrophotometer. Pyrogen-free water, used to dissolve the protein, was used as negative control.

### SDS–PAGE

SARS-CoV-2 S protein (1 µg) was diluted in loading buffer and loaded on 10%–20% Novex Tricine precast gel (Invitrogen) for electrophoresis at 120 V for 1 h. The gel was stained by using Coomassie Brilliant blue (Invitrogen). The image was obtained using a Gel Doc Imager (Bio-Rad Laboratories).

### BN-PAGE

SARS-CoV-2 S protein (2 µg) was incubated with 0.1, 0.25, or 0.5 mg/ml *E. coli* LPS or Lipid A for 30 min at 37°C in 20 µl as final volume. At the end of the incubation, the samples were separated under native conditions on BN-PAGE (Native PAGE BisTris Gels System 4%–16%, Invitrogen) according to the manufacturer’s instructions. Proteins were visualized by Coomassie staining. For western blotting, the material was subsequently transferred to a PVDF membrane using the Trans-Blot Turbo (Bio-Rad). Primary antibodies against the His-Tag (1:2000, Invitrogen) were followed by secondary HRP-conjugated antibody (1:2000, Dako) for detection of S protein. The protein was visualized by incubating the membrane with SuperSignal West Pico Chemiluminescent Substrate (Thermo Scientific) for 5 min followed by detection using a ChemiDoc XRS Imager (Bio-Rad). In another set of experiments, 2 µg SARS-CoV-2 S protein were incubated with 0.25 mg/ml LPS and Lipid A from *E. coli*, LPS from *P. aeruginosa*, LTA and PGN from *Staphylococcus aureus*, and zymosan from *Saccharomyces cerevisiae*, respectively. BN-PAGE and western blotting were performed as described above. LPS from *E. coli* and *P. aeruginosa* as well as Lipid A were purchased from Sigma-Aldrich, whereas LTA, PGN, and zymosan were purchased from InvivoGen. For the experiment described in Supplementary Figure S2C, we incubated 20 μl of 0.74 μM SARS-CoV-2 S protein or SARS-CoV S protein with 250 μg/ml *E. coli* LPS or 20 μl of 1.48 μM human prothrombin with 500 μg/ml *E. coli* LPS for 30 min at 37°C. Then, 1 μg of each protein was loaded on BN-PAGE followed by western blotting as described above. For the detection of prothrombin, polyclonal rabbit antibodies against the C-terminal prothrombin epitope VFR17 (VFRLKKWIQKVIDQFGE; diluted 1:1000, Innovagen AB) followed by swine anti-rabbit HRP-conjugated antibodies (1:1000, Dako) were used (Papareddy et al., 2010). Densitometric analysis was performed on the ∼480 kDa S protein and ∼66 kDa prothrombin bands, respectively, by using Image Lab 6.1 software (Bio-Rad Laboratories). Membranes from three independent experiment were analyzed and statistical analysis was performed using unpaired *t*-test with GraphPad Prism software v.8.

### MS analysis

After separation by SDS–PAGE or BN-PAGE and Coomassie staining, bands in the gels were cut out and the digestion was performed according to Shevchenko et al. (2006). Briefly, the gel pieces were washed with water, and then mixed with 50 mM ammonium carbonate in 50% acetonitrile (ACN). Gel pieces were shrunk with 100% ACN and then reduced with 10 mM DTT for 30 min at 56°C. Alkylation was performed with 55 mM idoacetamide at room temperature (RT). Trypsin solution (10 ng/µl) was added to cover the gel pieces placed on ice and after 1 h, the samples were placed at 37°C for overnight digestion. The supernatant was acidified using 5% formic acid and then analyzed by MALDI MS or LC–MS/MS.

### MALDI MS analysis

For MALDI MS analysis, digested SARS-CoV-2 S protein samples were mixed with a solution of 0.5 mg/ml α-Cyano-4-hydroxycinnamic acid (CHCA) in 50% ACN/0.1% phosphoric acid solution directly on a stainless MALDI target plate. Subsequent MS analysis was performed on a MALDI LTQ Orbitrap XL mass spectrometer (Thermo Scientific). Full mass spectra were obtained by using the FT analyzer (Orbitrap) at 60000 resolution (at *m*/*z* 400). Recording of mass spectra was performed in positive mode with an 800–4000-Da mass range. The nitrogen laser was operated at 27 μJ with automatic gain control (AGC) off mode using 10 laser shots per position. Evaluation of the spectra was performed with Xcalibur v 2.0.7. software (Thermo-Fisher Scientific).

### LC–MS/MS

The LC–MS/MS detection was performed on HFX Orbitrap equipped with a Nanospray Flex ion source and coupled with an Ultimate 3000 pump (Thermo-Fischer Scientific). Peptides were concentrated on an Acclaim PepMap 100 C18 precolumn (75 μm × 2 cm, Thermo Scientific) and then were separated on an Acclaim PepMap RSLC column (75 μm × 25 cm, nanoViper, C18, 2 μm, 100 Å) with heating at 45°C for both columns. Solvent A (0.1% formic acid) and solvent B (0.1% formic acid in 80% ACN) were used to create a nonlinear gradient to elute the peptides. For the gradient, the percentage of solvent B increased from 4% to 10% in 20 min, increased to 30% in 18 min, increased to 90% in 2 min, and then kept it for a further 8 min to wash the columns.

The Orbitrap HFX instrument was operated in data-dependent acquisition (DDA) mode. The peptides were introduced into mass spectrometer via stainless steel Nano-bore emitter (OD 150 µm, ID 30 µm) with the spray voltage of 1.9 kV and the capillary temperature was set 275°C. Full MS survey scans for *m*/*z* 350‒1600 with a resolution of 1200000 were performed in the Orbitrap detector. The automatic gain control (AGC) target was set to 3 × 10^6^ with an injecting time of 20 ms. The most intense ions (up to 20) with charge states 2‒5 from the full-scan MS were selected for fragmentation in Orbitrap. MS2 precursors were isolated with a quadrupole mass filter set to a width of 1.2 *m*/*z*. Precursors were fragmented by collision-induced dissociation (CID) with a collision energy of 27%. The resolution was set at 15000 and the values for the AGC target and inject time were 2 × 10^3^ and 60 ms, respectively, for MS/MS scans. The duration of dynamic exclusion was set 15 sec and the mass tolerance window was 10 ppm. MS/MS data were acquired in centroid mode. MS/MS spectra were searched with PEAKS (version 10) against UniProt Homo Sapiens (version 2020_02). The 10 ppm precursor tolerance and 0.02 Da fragment tolerance were used as the MS settings. Trypsin was selected as enzyme with one missed cleavage allowance, methionine oxidation and deamidation of aspargine and glutamine were treated as dynamic modification, and carbamidomethylation of cysteine was treated as a fixed modification. Maximum of posttranslational modifications per peptide was 2.

### MST

MST was performed on a NanoTemper Monolith NT.115 apparatus (Nano Temper Technologies). SARS-CoV-2 S protein (40 μg) and recombinant hCD14 (100 μg) were labelled by Monolith NT Protein labelling kit RED–NHS (Nano Temper Technologies) according to the manufacturer’s protocol. Either 5 μl of 20 nM labelled SARS-CoV-2 S protein or 20 nM labelled hCD14 were incubated with 5 μl of increasing concentrations of LPS (250–0.007 μM) in 10 mM Tris, pH 7.4. Then, samples were loaded into standard glass capillaries (Monolith NT Capillaries, Nano Temper Technologies) and the MST analysis was performed (settings for the light-emitting diode and infrared laser were 80%). Results shown are mean values± SD of six measurements.

### Molecular docking and simulations

LPS and Lipid A were docked onto the structures of prefusion S ECD trimer in the open (PDB: 6VSB) (Wrapp et al., 2020) and closed (PDB: 6XR8) (Cai et al., 2020) states. Missing loops of S ECD were modelled using Modeler version 9.21 (Sali and Blundell, 1993) and the best models were chosen based on the lowest discreet optimized protein energy (DOPE) scores (Shen and Sali, 2006) and stereochemical assessments using Ramachandran analysis (Ramachandran et al., 1963). The initial coordinates for *E. coli* rough LPS (R1 core type) and Lipid A were obtained from the CHARMM-GUI LPS modeler (Lee et al., 2019). Unbiased docking was performed using Vina-Carb (Nivedha et al., 2016) with a grid box of dimension 13 × 13 × 13 nm covering the whole protein surface. All torsion angles in the ligands were treated as flexible and default values were used in the docking configurations. Ten poses were generated for each docking calculation to give a total of 40 poses. Each pose was then inspected using VMD (Humphrey et al., 1996) for residues found within 0.4 nm of the ligand, specifically basic residues around the phosphate groups and hydrophobic residues around the lipid tails, to choose the most probable binding site. Chosen poses were then subject to all-atom MD simulations for the closed state of the S protein ECD. The S ECD‒Lipid A/LPS complexes used the CHARMM36 forcefield parameters (Huang and MacKerell, 2013). The systems were solvated with TIP3P water molecules and 0.15 M NaCl salt, before being minimized and equilibrated following the standard CHARMM-GUI protocols (Lee et al., 2016). Two independent 200-ns simulations with different starting distributions of velocities were performed for each system using GROMACS 2018 (Abraham, 2015). The temperature of the system was maintained at 310 K, while the pressure was maintained at 1 atm, using the Nosé−Hoover thermostat (Nosé, 1984; Hoover, 1985) and Parrinello−Rahman barostat (Parrinello and Rahman, 1981), respectively. Coulombic interactions were measured using the smooth particle mesh Ewald (PME) method (Essmann et al., 1995), while the van der Waals interactions were cut off at 1.2 nm with a force-smoothing function applied between 1.0 and 1.2 nm. Constraints were applied to all covalent bonds involving hydrogen atoms using the LINCS algorithm (Hess, 1997) to allow for a 2-fs integration time step. Simulations were visualized in VMD and contact analysis was performed using GROMACS tools.

### NF-઺B activation assay

THP1-XBlue-CD14 reporter cells (InvivoGen) were seeded in 96-well plates in phenol red RPMI, supplemented with 10% (*v*/*v*) heat-inactivated FBS and 1% (*v*/*v*) antibiotic-antimycotic solution (180000 cells/well). Cells were treated with 2.5 ng/ml LPS (Sigma) with increasing concentrations (0.1‒10 nM) of SARS-CoV-2 S protein or with 5 nM SARS-CoV-2 S protein with increasing concentrations of LPS (0.25‒1 ng/ml). Then, the cells were incubated at 37°C for 20 h. At the end of incubation, the NF-κB activation was analyzed according to the manufacturer’s instructions (InvivoGen), i.e. by mixing 20 μl supernatant with 180 μl SEAP detection reagent (Quanti-BlueTM, InvivoGen), followed by absorbance measurement at 600 nm. For the experiments with SARS-CoV S protein, we incubated THP1-XBlue-CD14 reporter cells with 5 nM SARS-CoV S protein and a dose range of LPS (0.25‒1 ng/ml). After an incubation period of 20 h at 37°C, NF-κB activation was measured as described above. Data shown are mean values ± SEM obtained from at least four independent experiments all performed in triplicate.

### MTT assay

The cytotoxicity of the treatments was evaluated by adding 0.5 mM thiazolyl blue tetrazolium bromide to the cells remaining from NF-κB activation assay. After 2 h of incubation at 37°C, cells were centrifuged at 1000 *g* for 5 min and then the medium was removed. Subsequently, the formazan salts were solubilized with 100 µl of 100% DMSO (Duchefa Biochemie). Absorbance was measured at a wavelength of 550 nm. Cell survival was expressed as percentage of viable cells in the presence of different treatments compared with untreated cells. Lysed cells were used as positive control. Data shown are mean values ± SD obtained from at least four independent experiments all performed in triplicate.

### Blood assay

Fresh venous blood was collected in the presence of lepirudin (50 mg/ml) from healthy donors. The blood was diluted 1:4 in RPMI-1640-GlutaMAX-I (Gibco) and 1 ml of this solution was transferred to 24-well plates and stimulated with 0.05 or 0.1 ng/ml LPS in the presence or the absence of 5 nM SARS-CoV-2 S protein. After 24 h incubation at 37°C in 5% CO_2_, the plate was centrifuged for 5 min at 1000 *g* and then the supernatants were collected and stored at −80°C before analysis. The experiment was performed at least four times by using blood from different donors each time.

### ELISA

The cytokines TNF-α, IL-1β, and IL-6 were measured in human plasma obtained after the blood experiment described above. The assay was performed by using human inflammation DuoSet^®^ ELISA Kit (R&D Systems) specific for each cytokine, according to the manufacturer’s instructions. Absorbance was measured at a wavelength of 450 nm. Data shown are mean values ± SEM obtained from at least four independent experiments all performed in duplicate.

### PBMC isolation

Fresh venous blood was collected in tubes with sodium citrate. Then, 1.5 part of blood was layered on 1 part of Polymorphprep (Fisher Scientific) before centrifugation at 600 *g* at RT for 35 min (without brakes). The layer containing PBMCs was collected in a new tube, diluted 1:1 (*v*/*v*) in PBS and centrifuged at 500 *g* at RT for 5 min. The supernatant was discarded, and the cells were resuspended in Erythrocyte Lysis Buffer (eBioscience). After 10 min incubation at RT, PBMCs were centrifuged for 5 min, washed once in RPMI (without phenol red) supplemented with 10% (*v*/*v*) heat-inactivated FBS and 1% (*v*/*v*) antibiotic–antimycotic solution, and resuspended in the same media at 1 × 10^6^ cells/ml. Cells were plated in a 96-well plate at a concentration of 180000 cells/well. The PBMCs were then treated with LPS and S protein as described above for the experiment with human blood. The supernatant was collected after 8 and 24 h and stored at −80°C before analysis by Luminex multiple bead assay. The experiment was performed three times using duplicate samples and blood from different donors each time.

### Luminex multiplex bead assay

The levels of TNF-α, IL-6, IL-8, IFN-β, IL-1β, CCL5, and IL-10 released by PBMCs in the supernatant after 8 and 24 h, respectively, were analyzed using a custom Human Magnetic Luminex^®^ Assay—human XL cytokine discovery pre-mixed kit (R&D Systems) according to the manufacturer’s protocol. The fluorescence was measured using a Luminex^®^ MAGPIX^®^ analyzer. The supernatants from duplicate samples of three independent experiments were analyzed. Data shown in Figure 3D represent mean of fold increase over control. The raw mean values ± SEM are given in Supplementary Table S1.

### Mouse inflammation model

The immunomodulatory effects of 5 µg SARS-CoV-2 S protein in combination or not with 2 µg LPS/mouse were analyzed employing BALB/c Tg(NF-κB-RE-Luc)-Xen reporter mice (Taconic Biosciences, 10–12 weeks old). The dorsum of the mouse was shaved carefully and cleaned. SARS-CoV-2 S protein was mixed with LPS immediately before subcutaneous injection on the dorsums of the mice anesthetized with isoflurane (Baxter). Then the animals were transferred to individually ventilated cages and imaged at 1, 3, and 6 h after the injection. An In Vivo Imaging System (IVIS Spectrum, PerkinElmer Life Sciences) was used for the longitudinal determination of NF-κB activation. Fifteen minutes before the IVIS imaging, mice were intraperitoneally given 100 µl D-luciferin (150 mg/kg body weight). Bioluminescence from the mice was detected and quantified using Living Image 4.0 Software (PerkinElmer Life Sciences).

### DLS

The hydrodynamic radii of *E. coli* LPS at increasing doses (100‒500 µg/ml) alone or with S protein (1.48 µM) were measured using a DynaPro Plate reader (WYATT Technology) equipped with a temperature-controlled chamber. S protein alone was used for control. The samples were incubated in a 384-well plate at 37°C for 30 min prior the analysis. Each measurement was performed 10 times. The hydrodynamic radii were analyzed using Dynamics 7.19 Software. The results are expressed as mean values ± SD obtained from three independent experiments. In another set of experiments, 500 µg/ml LPS were incubated with different concentrations of S protein (5‒250 nM) at 37°C for 30 min. The measurements were performed as described above.

### TEM analysis

Different concentrations of *E. coli* LPS (100‒500 µg/ml) alone or with S protein (1.48 µM) were used to prepare the samples for TEM. In brief, 5 μl of each sample were adsorbed onto carbon-coated grids (Copper mesh, 400) for 60 sec and stained with 7 μl of 2% uranyl acetate for 30 sec. The grids were rendered hydrophilic via glow discharge at low air pressure before using (Petrlova et al., 2020). Analysis was done on 15 view fields (magnification 4200×) of the mounted samples on the grid (pitch 62 μm) from three independent experiments.

### Effect of SARS-CoV-2 S protein on LPS-FITC aggregation

LPS-FITC (5 µg/ml; Sigma) was incubated with increasing concentrations of S protein (0.0074‒8880 nM) and then analyzed by recording the emission fluorescence spectra for 500‒600 nm, following excitation at 488 nm. All the measurements were performed using a Jasco J-810 spectropolarimeter equipped with an FMO-427S fluorescence module, with a scan speed of 200 nm/min and 2 nm slit width. The temperature was set to 25°C. The changes in the emission of FITC-LPS as a function of change in the aggregation state of LPS endotoxin-free water was monitored at 515 nm, as reported previously (Srivastava et al., 2012; Srivastava and Ghosh, 2013). The experiment was performed three times.

### Ethics statement

All animal experiments are performed according to Swedish Animal Welfare Act SFS 1988:534 and were approved by the Animal Ethics Committee of Malmö/Lund, Sweden. The use of human blood was approved by the Ethics Committee at Lund University, Lund, Sweden (permit no. 657-2008).

### Statistical analysis

All *in vitro* assays were repeated at least three times unless otherwise stated. Data are presented as means ± SEM or means ± SD. Differences in the mean between two groups were analyzed using Student’s *t*-test for normally distributed data and Mann–Whitney test otherwise. To compare means between more than two groups, a one-way ANOVA with Dunnett’s or Holm-Sidak posttest, or a two-way ANOVA with Sidak’s multiple comparisons tests, was used. Statistical analyses, as indicated in each figure legend, were performed using GraphPad Prism software v8. *P*-values <0.05 were considered to be statistically significant.

## Supplementary material

Supplementary material is available at *Journal of Molecular Cell Biology* online.

## Supplementary Material

mjaa067_Supplementary_Data
