# Supplementary material for: SARS-CoV-2 spike protein binds to bacterial lipopolysaccharide and boosts proinflammatory activity
Source: J Mol Cell Biol. 2020 Dec 9;12(12):916–32. doi: 10.1093/jmcb/mjaa067 (PMC7799037; doi:10.1093/jmcb/mjaa067)
Supplement: mjaa067_Supplementary_Data [file mjaa067_supplementary_data.pdf]

Supplementary material

SARS-CoV-2 Spike protein binds to bacterial lipopolysaccharide and boosts proinflammatory activity

Ganna Petruk[1,†], Manoj Puthia[1,†], Jitka Petrlova[1], Firdaus Samsudin[2], Ann-Charlotte Strömdahl[1], Samuel Cerps[3], Lena Uller[3], Sven Kjellström[4], Peter J. Bond[2,5], and Artur Schmidtchen[1,6,7,*]

[1] Division of Dermatology and Venereology, Department of Clinical Sciences, Lund University, SE-22184 Lund, Sweden

[2] Bioinformatics Institute (BII), Agency for Science, Technology and Research (A*STAR), Singapore 138671, Singapore

[3] Unit of Respiratory Immunopharmacology, Department of Experimental Medicine, Lund University, SE-22184 Lund, Sweden

[4] Division of Mass Spectrometry, Department of Clinical Sciences, Lund University, SE-22184 Lund, Sweden

[5] Department of Biological Sciences, National University of Singapore, Singapore 117543, Singapore

[6] Copenhagen Wound Healing Center, Bispebjerg Hospital, Department of Biomedical Sciences, University of Copenhagen, DK-2400 Copenhagen, Denmark

[7] Dermatology, Skåne University Hospital, SE-22185 Lund, Sweden

[†] These authors contributed equally to this work.

* Correspondence to: Artur Schmidtchen, Tel.: +46 462224522; E-mail: artur.schmidtchen@med.lu.se

Supplementary Figures

A

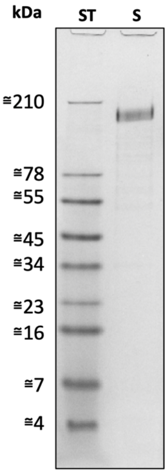

B

```
VNLTTRTQLP PAYTNSFTRG VYYPDKVFRS SVLHSTQDLF LPFFSNVTWF HAIHVSGTNG TKRFDNPVLP
FNDGVYFAST EKSNIIRGWI FGTTLDSKTQ SLLIVNNATN VVIKVCEFQF CNDPFLGVYY HKNNKSWMES
EFRVYSSANN CTFEYVSQPF LMDLEGKQGN FKNLREFVFK NIDGYFKIYS KHTPINLVRD LPQGFSALEP
LVDLPIGINI TRFQTLLALH RSYLTPGDSS SGWTAGAAAY YVGYLQPRTF LLKYNENGTI TDAVDCALDP
LSETKCTLKS FTVEKGIYQT SNFRVQPTES IVRFPNITNL CPFGEVFNAT RFASVYAWNR KRISNCVADY
SVLYNSASFS TFKCYGVSPT KLNDLCFTNV YADSFVIRGD EVRQIAPGQT GKIADYNYKL PDDFTGCVIA
WNSNNLDSKV GGNYNYLYRL FRKSNLKPFE RDISTEIYQA GSTPCNGVEG FNCYFPLQSY GFQPTNGVGY
QPYRVVVLSF ELLHAPATVC GPKKSTNLVK NKCVNFNFNG LTGTGVLTES NKKFLPFQQF GRDIADTTDA
VRDPQTLEIL DITPCSFGGV SVITPGTNTS NQVAVLYQDV NCTEVPVAIH ADQLTPTWRV YSTGSNVFQT
RAGCLIGAEH VNNSYECDIP IGAGICASYQ TQTNSPRAAA SVASQSIIAY TMSLGAENSV AYSNNSIAIP
TNFTISVTTE ILPVSMTKTS VDCTMYICGD STECSNLLLQ YGSFCTQLNR ALTGIAVEQD KNTQEVFAQV
KQIYKTPPIK DFGGFNFSQI LPDPSKPSKR SFIEDLLFNK VTLADAGFIK QYGDCLGDIA ARDLICAQKF
NGLTVLPPLL TDEMIAQYTS ALLAGTITSG WTFGAGAALQ IPFAMQMAYR FNGIGVTQNV LYENQKLIAN
QFNSAIGKIQ DSLSSTASAL GKLQDVVNQN AQALNTLVKQ LSSNFGAISS VLNDILSRLD KVEAEVQIDR
LITGRLQSLQ TYVTQQLIRA AEIRASANLA ATKMSECVLG QSKRVDFCGK GYHLMSFPQS APHGVVFLHV
TYVPAQEKNF TTAPAICHDG KAHFPREGVF VSNGTHWFVT QRNFYEPQII TTDNTFVSGN CDVVIGIVNN
TVYDPLQPEL DSFKEELDKY FKNHTSPDVD LGDISGINAS VVNIQKEIDR LNEVAKNLNE SLIDLQELGK
YEQYIKWP
```

Figure S1 SARS-CoV-2 S protein sequence and endotoxin content. (**A**) 1 µg of SARS-CoV-2 S protein was separated by SDS-PAGE (16.5% Tris-Tricine gel) followed by Coomassie staining. (**B**) LC-MS/MS data were obtained after in gel digestion of SARS-CoV-2 S protein after SDS-PAGE separation. Database analysis confirmed the identity of the recombinant protein to SARS-CoV-2 S protein identifying 56% of the protein sequence as shown in red. Totally 110 peptides correspond to the protein sequence, two other proteins were detected i.e. keratin type II (25% sequence coverage, 14 unique peptides) and keratin type I (9% sequence coverage, 5 unique peptides).

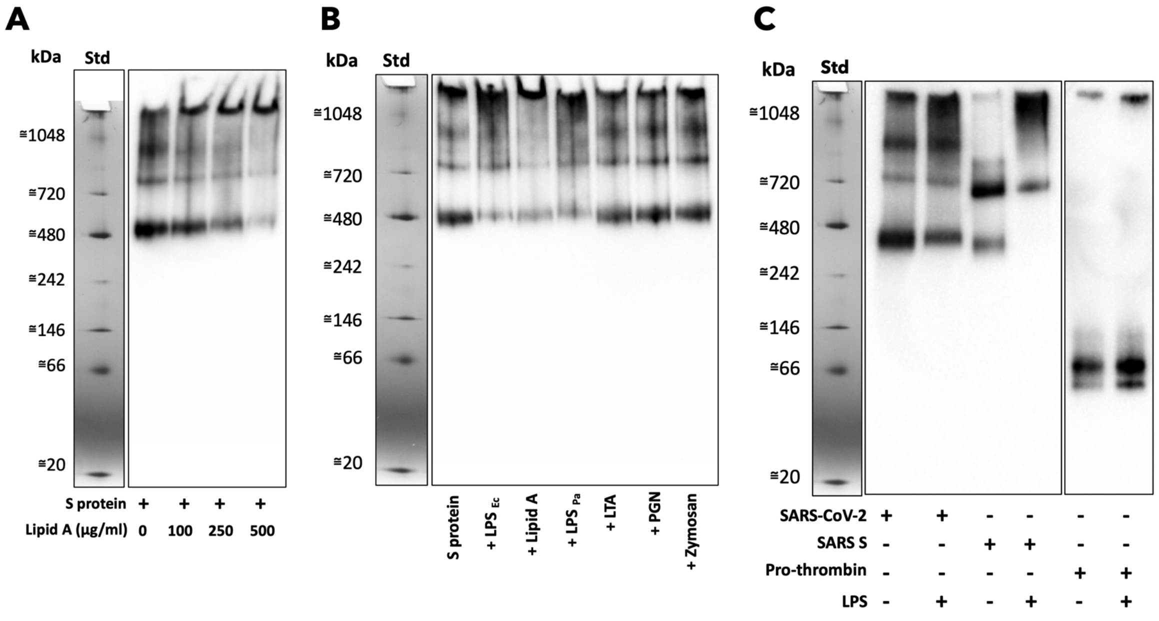

Figure S2 Analysis of binding of SARS-CoV-2 S protein to different TLR ligands or of SARS-CoV S protein to LPS. BN-PAGE followed by Western blotting of (**A**) SARS-CoV-2 S protein incubated with 0-0.5 mg/ml of Lipid A; (**B**) SARS-CoV-2 S protein incubated with 0.25 mg/ml of LPS from *E. coli* (LPS $_{Ec}$), Lipid A, LPS from *P. aeruginosa* (LPS $_{Pa}$), LTA, PGN and zymosan; (**C**) SARS-CoV-2 S protein, SARS-CoV S protein (SARS S) and prothrombin with *E. coli* LPS (0.74 M : 250 µg/ml, protein : LPS).

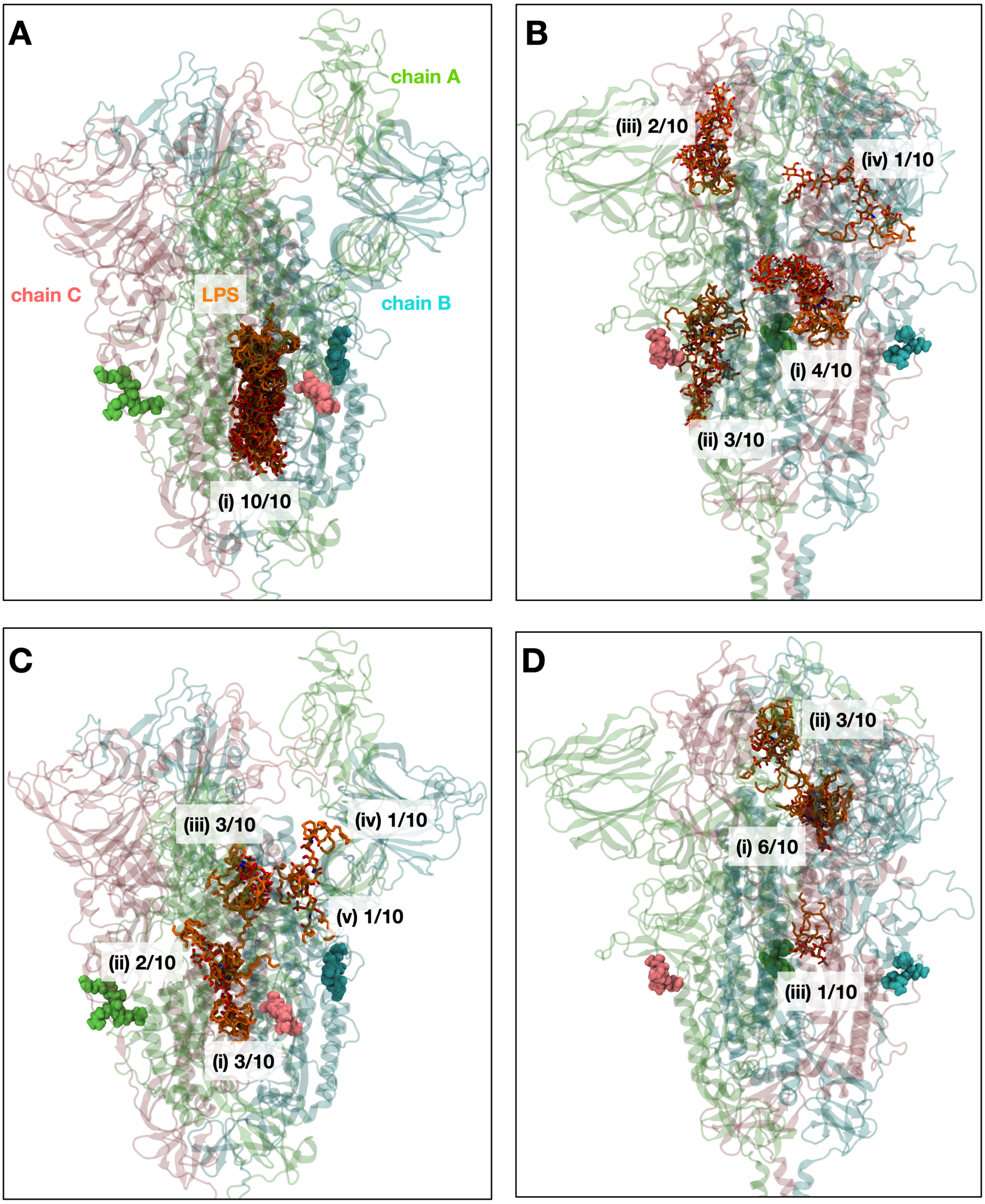

Figure S3 The binding poses generated by flexible docking of *E. coli* LPS and Lipid A on S ECD. (**A**) Docking of LPS (orange; stick representation) onto S ECD in open conformation (green, cyan and pink; cartoon representation). (**B**) Docking of LPS onto S ECD in closed conformation. (**C**) Docking of Lipid A onto S ECD in open conformation. (**D**) Docking of Lipid A onto S ECD in closed conformation. The number of docking poses for each binding site (out of 10 total poses in each docking calculation) are shown. The S1/S2 furin cleavage sites are highlighted in sphere representation.

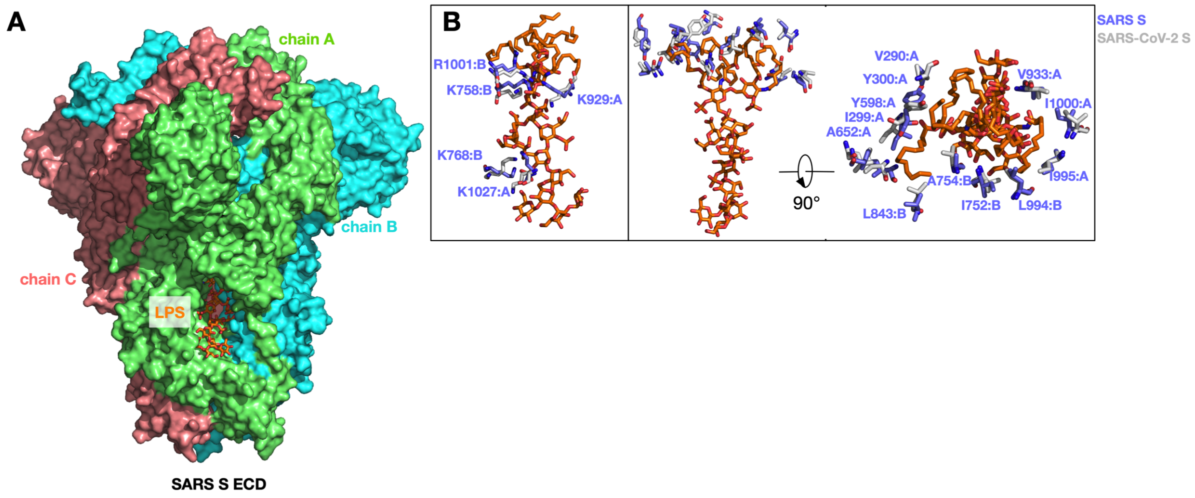

Figure S4 Comparison of SARS-CoV-2 and SARS-CoV S proteins. (**A**) The cryo-EM structure of SARS-CoV S ECD (PDB: 5X58) was aligned to the structure of SARS-CoV-2 S ECD (PDB: 6VSB) and the docked LPS molecule from the latter was mapped to the former. (**B**) The conservation of residues important for binding in the proposed LPS binding site. Residues from SARS-CoV S are shown in purple and labelled, while corresponding residues from SARS-CoV-2 S are shown in white.

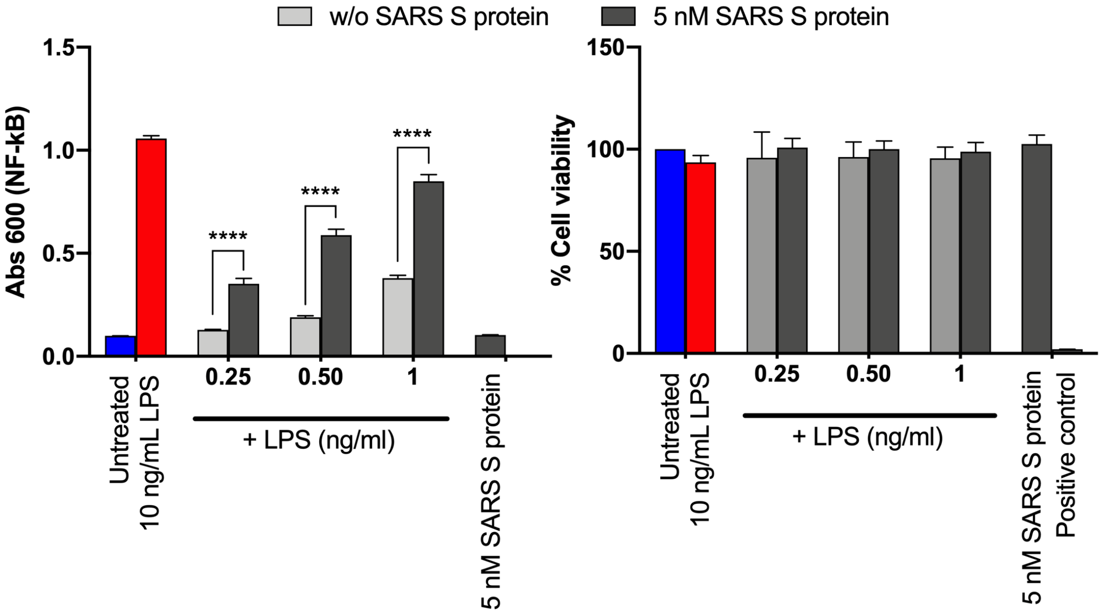

Figure S5 Effects of SARS-CoV S protein on LPS-induced responses in THP-1 cells. THP-1-XBlue-CD14 cells were treated with increasing concentrations of LPS (0.25-1 ng/ml) and 5 nM SARS-CoV S protein (SARS S) then the NF-κB activation was measured. The viability of THP-1 cells after treatment with SARS S protein and LPS was measured by MTT. The mean ± SEM (NF-kB) or SD (MTT assay) values of four independent experiments performed in triplicate are shown (n=4). ****, $P < 0.0001$, determined using two-way ANOVA with Sidak´s multiple comparisons test (NF-kB) or one-way ANOVA with Dunnett’s multiple comparison test (MTT assay). na; not analyzed, w/o; without.

Supplementary Table

Table S1. Data for cytokines released from human PBMCs.

	Cytokine/ Chemokine (pg/ml)	Mean ± SEM<br>0.05 ng/ml LPS<br>-	 <br>+ 5 nM S protein	*P* value	Mean ± SEM<br>0.1 ng/ml LPS<br>-	 <br>+ 5 nM S protein	*P* value
8 h	**TNF-α**	1289 ± 218.2	2824 ± 311.2	**0.0387**	2204 ± 350.7	3913 ± 392.3	**0.0182**
	**IL-6**	5328 ± 1354	10651 ± 1876	**0.0336**	9306 ± 1690	14316 ± 1512	**0.0164**
	**IL-1β**	262.3 ± 63.6	776.4 ± 185.3	0.1747	560 ± 112.7	1326 ± 264.9	0.1445
	**IL-8**	3935 ± 165.2	4088 ± 96.2	0.7571	4171 ± 36.0	4257 ± 54.5	0.7953
	**IFN-β**	2.1 ± 0.0	4.5 ± 0.5	0.1034	2.3 ± 0.1	4.4 ± 0.1	**< 0.0001**
	**CCL5**	557.5 ± 160.1	682.9 ± 115	0.6378	647.1 ± 118.4	647 ± 114.2	> 0.9999
	**IL-10**	56.28 ± 5.0	138 ± 18.12	0.0926	100.8 ± 10.6	191.4 ± 28.3	0.1999
24 h	**TNF-α**	1147 ± 141.2	2379 ± 259	**0.0423**	2050 ± 602	3365 ± 413.3	0.2796
	**IL-6**	7732 ± 1885	14142 ± 1995	**0.0124**	11463 ± 3353	16013 ± 2940	0.0818
	**IL-1β**	307.7 ± 78.8	992.7 ± 205.1	0.1295	655.1 ± 218.2	1636 ± 313.8	0.1133
	**IL-8**	4356 ± 63.8	3655 ± 716.1	0.8811	3854 ± 621.1	4403 ± 48.4	0.9247
	**IFN-β**	2.0 ± 0.1	3.0 ± 0.1	**0.0404**	2.0 ± 0.1	3.1 ± 0.1	0.0563
	**CCL5**	496 ± 124.6	645.2 ± 132.4	0.3902	565 ± 159.6	716.6 ± 156.5	0.6499
	**IL-10**	124.4 ± 28.76	817.4 ± 264.6	0.3199	290.4 ± 102	1441 ± 435.4	0.4620

P values were determined using a one-way ANOVA with Tukey's post test. LPS + S protein groups are compared with their respective LPS alone groups (-). *P* values <0.05 are in bold text.
